# Graphene Based Surface Plasmon Polariton Modulator Controlled by Ferroelectric Domains in Lithium Niobate

**DOI:** 10.1038/srep18258

**Published:** 2015-12-14

**Authors:** Hao Wang, Hua Zhao, Guangwei Hu, Siren Li, Hang Su, Jingwen Zhang

**Affiliations:** 1 Institute of Modern Optics, Department of Physics, Harbin Institute of Technology, Harbin, 150001, China; 2 Key Laboratory of Micro-Optics and Photonics Technology of Heilongjiang Province, Harbin, 150001, China

## Abstract

We proposed a ferroelectric domain controlled graphene based surface plasmon polariton modulator. Ferroelectricity-induced electronic and optical property tuning of graphene by domain in lithium niobate was theoretically investigated considering both interband and intraband contributions of surface conductivity. With the corrected Sellmeier equation of lithium niobate, the propagation of transverse magnetic mode surface plasmon polaritons in an air/graphene/lithium niobate structure was studied when monolayer graphene was tuned by down polarization direction ferroelectric domain with different polarization levels. The length of the ferroelectric domain was optimized to be 90 nm for a wavelength of 5.0 μm with signal extinction per unit 14.7 dB/μm, modulation depth 474.1 dB/μm and figure of merit 32.5. This work may promote the study of highly efficient modulators and other ultra-compact nonvolatile electronic and photonic devices in which two-dimensional materials and ferroelectric materials are combined.

As a two-dimensional (2D) electron system with a monolayer of carbon atoms arrayed in a honeycomb lattice, graphene possesses a linear dispersion relation, and the extraordinary properties of the chiral fermions make it a promising platform for electronic and photonic applications^1^. Nowadays, it has attracted increasing attention due to its unique optical and electronic properties, such as fine structure-defined optical transmission, anomalous quantum Hall effects, chiral tunneling, and ultra-high mobility of carriers (~10^6^ m/s). The most thriving research field in which graphene is being studied is plasmonics, which describes the interaction between electron oscillations and electromagnetic field^2^,^3^,^4^,^5^, in that surface plasmons excited in graphene are much more confined than those in conventional noble metals. Moreover, its low loss and flexible nature make graphene a potential alternative material for plasmonic applications^6^. Since the chemical potential of graphene is particularly influenced by carrier density, various doping methods have been used to tune graphene’s optical and electric properties, for instance, chemical doping^7^, photo-induced doping^8^,^9^, substrate-contact doping^10^, and gate electric field tuning^11^. The combination of graphene with ferroelectric materials has been intensively studied in recent years because the remanent ferroelectric polarization can drive carrier modulation in graphene via electrostatic doping^12^, which has promising applications in stable hysteresis curves^13^, field-effect transistors (FETs)^14^, nonvolatile memory^15^, and flexible transparent electronic devices^16^. Recently, Baumer C. *et al.* researched a graphene growth and transfer method on periodically poled lithium niobate (PPLN)^17^,^18^, and discussed graphene carrier density modulation with Kronig-Penney type potential, interface chemical process and density functional theory (DFT) calculations to determine the net surface-bound charge of the ferroelectric material^19^. The ability to create spatially tuned *p-n* junctions in graphene makes ferroelectric materials potential alternatives to gate electrodes. Single-layer molybdenum disulfide (MoS_2_, a typical direct bandgap semiconductor) on PPLN also has been studied for the convenience of ferroelectric polarization pattern fabrication without need of lithography^20^. Additionally, motivated by the recent developments in nanometer-scale ferroelectric domain growth technology and the future in data storage, information processing, and photonic devices^21^,^22^,^23^,^24^,^25^, we theoretically investigated the graphene surface plasmon polariton (SPP) modulator controlled by ferroelectric domains in congruent grown lithium niobate (CLN). Tuning of the electronic and optical properties of graphene induced by ferroelectricity was discussed when considering both interband and intraband transitions from the visible range to mid-wave infrared (MWIR) range. The SPP wave propagating distance and lateral penetration length were also studied with different chemical potentials and wavelengths. The length of the ferroelectric domain was optimized to be 90 nm for wavelength at 5.0 μm considering the signal extinction, modulation depth and power ratio. The combination of lithium niobate (LN, renowned as “optical silicon”) with 2D materials may lead to new nonvolatile integration devices, plasmonic modulators, and other ultra-compact electronic and photonic devices.

## Results

### Ferroelectricity-induced optical property tuning of graphene

**Figure 1:**
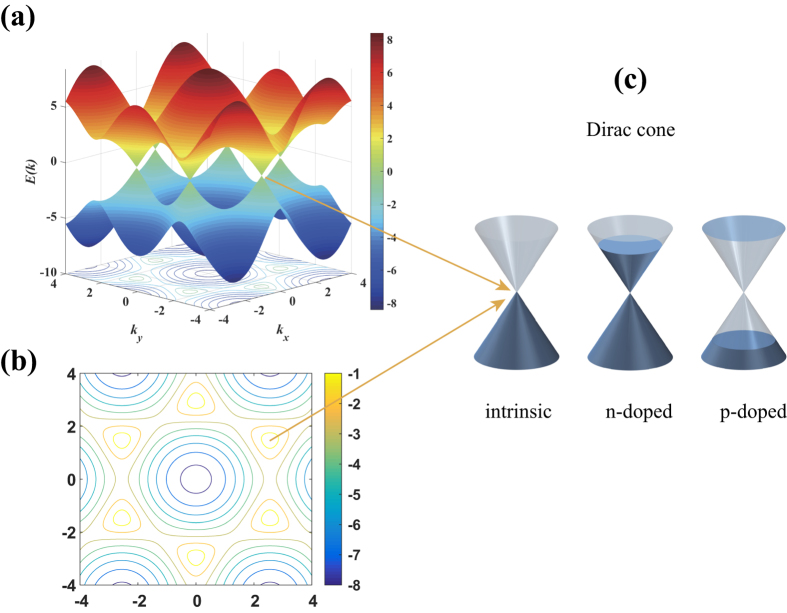
Linear dispersion relation of graphene. (**a**) Band structure of monolayer graphene. (**b**) Contour plot of band structure in (a). (**c**) Magnified Dirac cones in different doping levels (intrinsic, n-doped, and p-doped).

**Figure 2:**
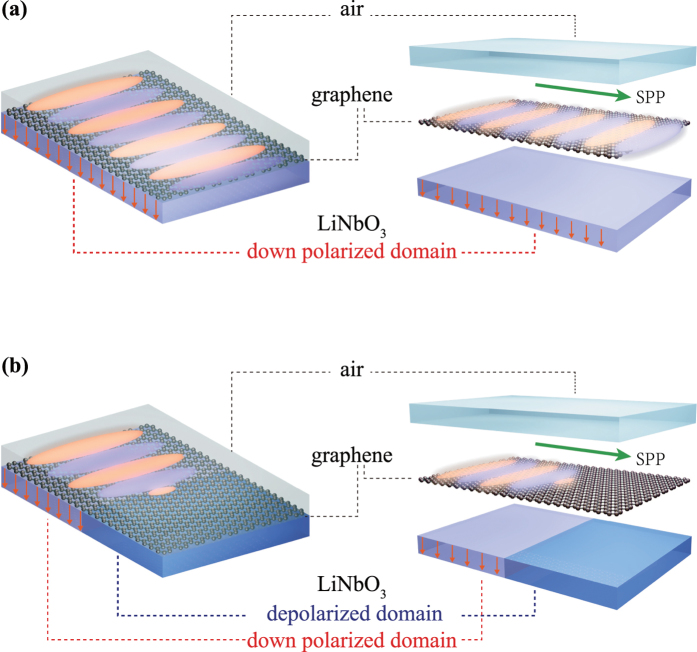
Schematic of modulator structure with pristine monolayer graphene on LN. (**a**) Graphene on a uniformly polarized domain of a CLN substrate. (**b**) Graphene on a CLN substrate with different ferroelectric polarized domains. The arrows represent the down-polarized domain and the remaining part represents the depolarized domain. Red and blue patterns represent the SPP propagating in (**a**) and being stopped by the depolarized domain in (**b**). The green arrow represents the propagation direction of the SPPs. The purposely separated parts of the air/graphene/CLN structure on the right side show the strong confinement of SPPs in graphene.

**Figure 3:**
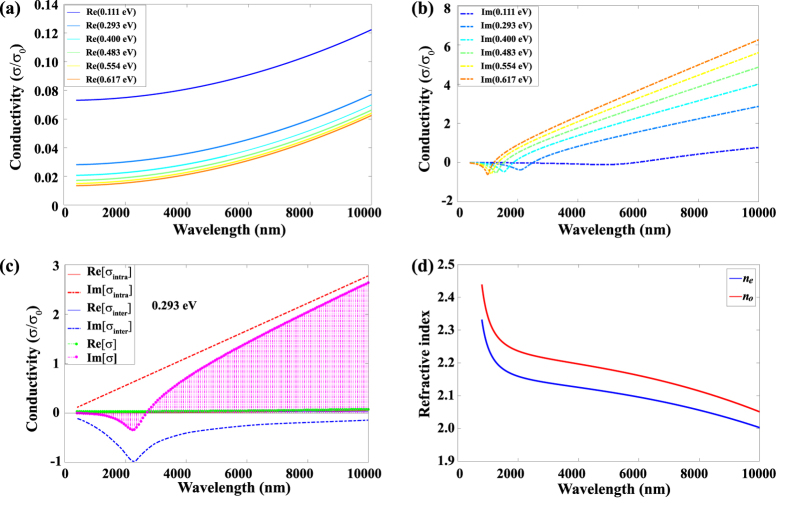
Surface conductivity of p-doped monolayer graphene and anisotropic refractive indices of CLN. (**a**,**b**) show the real and imaginary parts of the surface conductivity of p-doped monolayer graphene at different chemical potentials (*μ*_*c*_ = 0.111 eV, 0.293 eV, 0.400 eV, 0.483 eV, 0.554 eV, and 0.617 eV, corresponding to carrier concentrations of 1 × 10^12^ cm^−2^, 7 × 10^12^ cm^−2^, 1.3 × 10^13^ cm^−2^, 1.9 × 10^13^ cm^−2^, 2.5 × 10^13^ cm^−2^, and 3.1 × 10^13^ cm^−2^, respectively). (**c**) Real and imaginary parts of interband conductivity and intraband conductivity of graphene at 0.293 eV. The stemmed green and magenta lines are the total real and imaginary parts of surface conductivity, respectively. (**d**) Extraordinary (blue line) and ordinary (red line) refractive indices of CLN.

**Figure 4:**
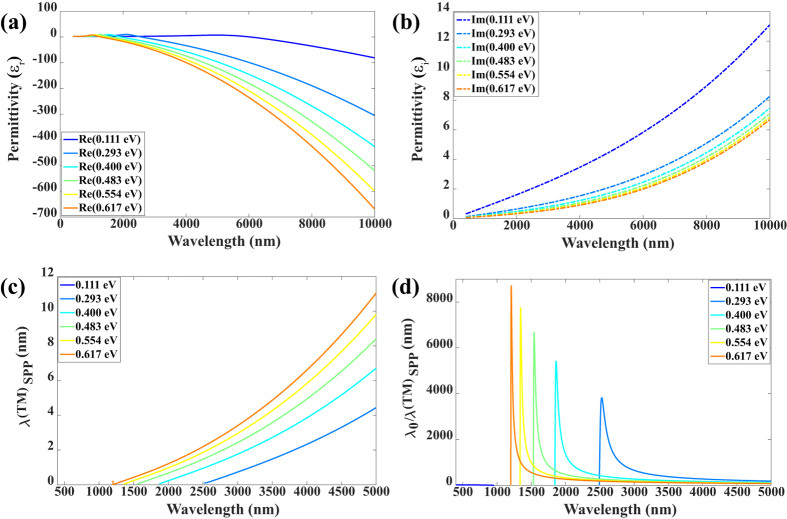
Permittivity of the p-doped monolayer graphene and confinement of SPP in the air/graphene/CLN structure. (**a**,**b**) The real and imaginary parts of the permittivity of the p-doped monolayer graphene at different chemical potentials (see corresponding carrier concentration in Fig. 3). (**c**) Confinement of SPP in the air/graphene/CLN structure at different chemical potentials. (**d**) Curves obtained by comparing the incident wavelength and lateral penetration depth at different chemical potentials.

The band structure (or energy dispersion) of monolayer graphene with six Dirac cones (*K* points) at the corners of the 2D hexagonal Brillouin zone is depicted in Fig. 1(a,b). Electrons and holes near the Dirac points behave like relativistic particles with 1/2 spin due to the linear dispersion relation. The unique properties of these Dirac fermions make graphene a zero-gap semiconductor, quite different from conventional three-dimensional (3D) semiconductors. Besides, the electronic and optical properties of graphene are mainly controlled by the chemical potential *μ*_*c*_, i.e., Fermi energy level *E*_*F*_. With random-phase approximation (RPA) under the self-consistent-field linear response theory, the surface conductivity of graphene can be derived from the Kubo formula consisting of both interband and intraband transitions^1^ as follows:


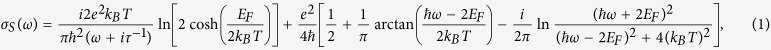


where *k*_*B*_ is the Boltzmann constant, *T* is the room temperature, *ħ* is the reduced Planck constant, *ω* is the angular frequency, and *τ* is the carrier relaxation lifetime, defined as *μμ*_*c*_*/eν*_*F*_^2^. The first term is attributed to intraband transition and the second term is attributed to interband transition. For different carrier densities, the surface conductivity varies with chemical potential because *μ*_*c*_ is determined by the following equation:


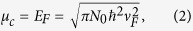


where *N*_*0*_ is the carrier concentration of graphene and *ν*_*F*_ is the Fermi velocity (see Supplementary Table S1 online for the detailed parameters used in the calculation). When the pristine graphene^26^,^27^,^28^ was transferred to the LN surface (Fig. 2), contact with up or down ferroelectric domains of LN induced n-type or p-type doping in the graphene, with carriers electrons or holes, respectively. Moreover, varying the polarization level leads to different net surface-bound charges (see Supplementary section 3 for spontaneous polarization in LN), resulting in the different surface conductivities of the graphene^19^. Here, we investigated p-doped monolayer graphene on CLN (48.38 mol.% lithium oxide), i.e., the CLN sample was polarized to retain the down polarization direction ferroelectric domain (represented by the orange arrows in Fig. 2). As shown in Fig. 3(a,b) (unit *e*^*2*^*/4ħ*), the real part of the surface conductivity decreases with increasing carrier concentration, while the imaginary part increases with carrier concentration and the valley (corresponding to 2*ħω/μ*_*c*_) shifts from the MWIR range to the near-infrared (NIR) wavelength range. Generally, the interband contribution dominates from the visible to the NIR range for slightly doped graphene, while the intraband process plays a more significant role in the mid-IR, and far-IR regions, including the terahertz(THz) regions^1^,^4^, which can be easily seen in Fig. 3(c). The permittivity tensor can be obtained from the uniaxial anisotropic permittivity function by converting the surface conductivity to the effective volume conductivity (*σ*_*V*_ = *σ*_*S*_/Δ),


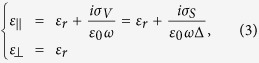


where Δ = 0.34 nm is thickness of monolayer graphene, 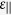 is the in-plane (parallel) component, 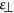 is the out-plane (perpendicular) component, and *ε*_*r*_ = 2.5 is the background relative permittivity. The in-plane component is shown in Fig. 4(a,b) with a giant negative value of the real part and a small positive imaginary part, indicating strong confinement and relative low loss of electromagnetic waves at the graphene surface. This can also be confirmed with the semi-classical model derived from the Kubo formula^27^,^29^ (see Supplementary section 2).

### SPP in the air/graphene/CLN structure

The anisotropic refractive indices of CLN can be obtained from the three-oscillator Sellmeier equation,


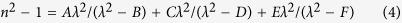


which incorporated two UV oscillators and an IR oscillator to correspond to the experimental data^30^ (see Supplementary section 4 for the coefficient values). To realize the maximum utilization of ferroelectric polarization, the *c*-axis (optical axis) is perpendicular to the graphene/CLN interface, i.e., the ordinary and extraordinary refractive indices (Fig. 3(d)) are considered as in-plane and out-plane values, respectively.

**Figure 5:**
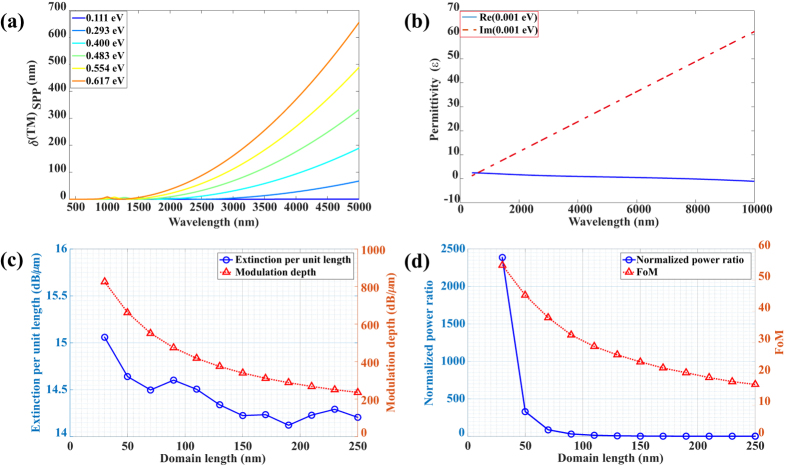
Optimization of length of the ferroelectric domain. (**a**) Propagation length of the SPP in the air/graphene/CLN structure. (**b**) Real and imaginary parts of the permittivity of the p-doped monolayer graphene at chemical potential *μ*_*c*_ = 0.001 eV, corresponding to a carrier concentration approximately 1 × 10^8^ cm^−2^). (**c**) Extinction per unit length (blue line with circular markers) and modulation depth (red line with triangular markers) of the air/graphene/CLN structure at a wavelength of 5.0 μm. (**d**) Power ratio (blue line with circular markers) and figure of merit (red line with triangular markers) of the air/graphene/CLN structure at wavelength of 5.0 m. The length of the polarized ferroelectric domain from 30 nm to 250 nm having an interval of 20 nm.

The transverse magnetic (TM) and transverse electric (TE) modes are supported in conventional materials that are used for optical fiber, waveguide, and other optical devices^31^,^32^,^33^. However, the TE mode cannot exist in the traditional 2D electron gas because the imaginary part of the conductivity is always positive^4^,^34^,^35^. In graphene, the TE mode can be supported because of its unique nature when Im(*σ*) is negative^29^,^35^,^36^,^37^. When Im(*σ*) <0, the wavelength shifts from the infrared to visible region when the chemical potential increases, which is calculated using the RPA model, i.e., into the high loss region where the SPP propagation length is quite small, as shown in Fig. 3(b). Consequently, the corresponding bandwidth becomes narrower. Therefore, we only consider the TM mode here^9^,^37^,^38^,^39^. The dispersion relation in the system can be written as^40^:


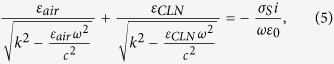


where, *ε*_*air*_ and *ε*_*CLN*_ are permittivities of air and CLN, respectively. From Fig. 4(c,d) we can observe that the lateral penetration depth (*λ(TM)*_*SPP*_) is much shorter than that in conventional noble metals, whose *λ(TM)*_*SPP*_ is usually hundreds of nanometers even though at visible range^41^. However, the propagation length (*δ(TM)*_*SPP*_) (Fig. 5(a)) makes this structure less applicable for waveguides that can be improved by higher doping level of graphene for longer wavelength. The penetration depth and propagation length increase with higher chemical potential, i.e., stronger polarization of the ferroelectric domain. Hence, a trade-off between these evaluation parameters needs to be considered for different applications.

### Optimization of length of the ferroelectric domain

As the absorption of SPP in a graphene modulator depends strongly on the chemical potential, a depolarized ferroelectric domain of CLN could be used to tune the graphene into a quasi-neutral state^9^ with *μ*_*c*_ of approximately 0.001 eV, which corresponds to a very small carrier concentration of approximately 1 × 10^8^ cm^−2^. Figure 5(b) shows that the real part of the permittivity at 0.001 eV is positive, and hence the TM mode cannot be supported. Furthermore, the imaginary part of the permittivity is quite large, which means strong absorption of the corresponding energy of the electromagnetic wave. Here we consider a typical wavelength of 5.0 μm in the MWIR range with lateral penetration length and propagation length of approximately about 11 nm (Fig. 4(c)) and 675 nm (Fig. 5(a)), respectively. The chemical potential of graphene on a uniformly down-polarized ferroelectric domain is selected as 0.617 eV, i.e., the permittivity of the p-doped graphene and quasi-neutral is −161.7 + 1.384i and 0.8129 + 57.26i, respectively. The corresponding ordinary and extraordinary refractive indices of CLN are 2.051 and 2.003, respectively.

**Figure 6:**
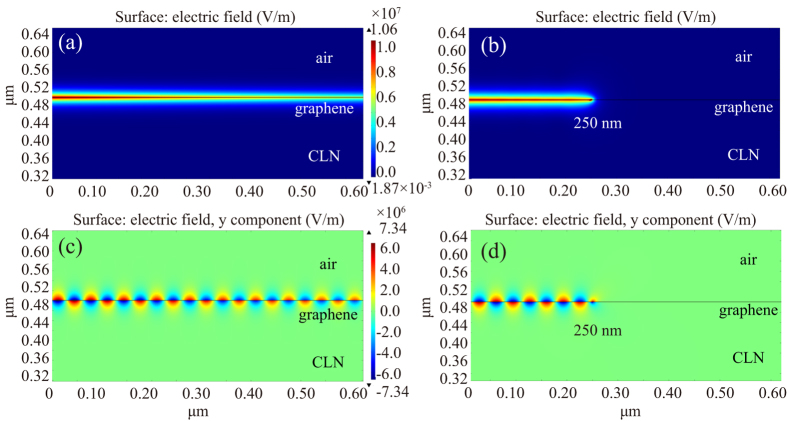
Surface electric field of the graphene modulator with different length of ferroelectric domain at a wavelength of 5.0 μm (side view). (**a**) Surface electric field of graphene on the CLN substrate with uniformly polarized ferroelectric domain. (**b**) Surface electric field of graphene on the CLN substrate with 250 nm down-polarized ferroelectric domain. (**c**) The corresponding *y*-component of the surface electric field. (**d**) The corresponding *y*-component of the surface electric field. The thin black line represents the monolayer graphene.

The energy of the TM mode is strongly confined near the graphene surface (Fig. 6(a) and (Fig. 6(c), corresponding to Fig. 2(a)) with ultra-high electric field intensity of approximately 1 × 10^7^ V/m and the SPP propagates at the interface, which is similar to the conventional insulator/metal/insulator (IMI) structure. Although the energy flow gets into the graphene structure on the depolarized ferroelectric domain, it is almost perfectly absorbed. In addition, the SPP will still propagate into the depolarized region and there is a short extinction length of SPP, which can be observed from Fig. 6(b) and (Fig. 6(d)(corresponding to Fig. 2(b)). To calculate the optimal length of the ferroelectric domain for SPP modulation, three factors associated with modulator performance are considered, which include the signal extinction per unit length, logarithmic extinction ratio per unit length (modulation depth), and figure of merit (FoM)^42^ (see Supplementary section 5 for the details). Here, we can define the domain-off (a ferroelectric domain period has a down-polarized domain and a depolarized domain with same length) and domain-on (a ferroelectric domain period has a down-polarized domain) states similar to voltage-off and voltage-on states in the traditional electro-optic modulator (also can be regarded as “0” and “1” states), and hence the direct power ratio of *P*_*off*_ and *P*_*on*_ (plasmonic power for domain-off and domain-on state, respectively) is also an vital factor for consideration. In this modulator, we observe that when the domain length varies from 250 nm to 30 nm, the modulation depth increases from 235.9 dB/μm to 825.3 dB/μm (Fig. 5(c)); meanwhile, FoM increases from 16.6 to 54.8. However, the value of extinction per unit length exhibits a slightly ascending trend from 14.2 to 15.1 with several drops at 190 nm and 70 nm, as shown in Fig. 5(d). Moreover, the normalized power ratio increases approximately 2600 times from 250 nm to 30 nm, whereas it is 40 for 90 nm. Considering these four factors, the optimized ferroelectric domain length should be around 90 nm for comparison and modulation of the on-off states. As compared to other modulators^9^,^11^,^43^,^44^, our structure can achieve relatively higher extinction per unit length and greater modulation depth based on the simulation. In addition, the SPP can excite intrinsic polaritons in the depolarized domain^45^,^46^,^47^, nevertheless, they do not affect the results for most of the energy is absorbed by graphene.

## Discussion

Chemical vapor deposition (CVD) is the most common method to grow graphene and then graphene can be transferred onto a ferroelectric surface. Consequently, the surface chemical reconstruction, O-derived defects, and temperature-dependent polarization will change the amount of ferroelectric surface-bound charge^19^, and hence the doping level of graphene. Further studies should be performed for the design of related applications. Moreover, the RPA model may still not perfectly describe the dispersion relation of graphene although it corresponds well with some experiments and is better than the semi-classical model; however, it is still the most widely used approach for simulation and experiments at the wavelength range from visible to infrared^39^,^48^,^49^,^50^,^51^,^52^,^53^,^54^. Other methods like tight-binding approximation (TBA), Dirac equation continuum model (DECM), and first-principle calculation (ab initio) can also correspond well with some experiments, especially for the acoustic-like plasmons in graphene at long wavelengths^4^.

In addition, other ferroelectric domain structures in different shapes^22^,^55^ can also be designed to realize energy control when combined with traditional semiconductors, such as indium-tin oxide^56^,^57^,^58^, or 2D materials, such as graphene, bi-layer graphene, and metal dichalcogenides MX_2_^20^. Moreover, this can stimulate the development of a new type of solar cell for energy harvesting with different types of inorganic, organic and hybrid perovskite structured materials^59^.

In conclusion, in this paper, we have proposed a graphene modulator controlled by the ferroelectric domain in LN. Based on the ferroelectricity-induced tuning of the electronic and optical properties of graphene, we can use domains with different ferroelectric polarization levels to realize propagation control of SPPs in the air/graphene/CLN structure. Furthermore, the domain length can be optimized by considering the signal extinction per unit length, modulation depth, normalized power ratio, and FoM. For the typical wavelength of 5.0 μm in the MWIR range, we obtained an optimized value of 90 nm. In addition, other ferroelectric materials, such as lead zirconate titanate and barium titanate, can be utilized in similar structures fabricated using 2D and ferroelectric materials. This work can promote the study of highly efficient modulators and other nonvolatile electronic and photonic devices for future on-chip applications.

## Methods

The simulation was performed using the commercial finite element method (FEM) software “COMSOL Multiphysics” and we used “Boundary Mode Analysis” module to obtain the TM mode in the air/graphene/CLN structure. The heights of the two-dimensional structure simulation of the three types of materials were 300 nm, 0.34 nm and 300 nm, respectively, and the length was 1200 nm for each material. To obtain accurate results, the graphene structure was vertically meshed into five layers having an interval length of 1 nm. The left boundary was set as the input port and the right one was the output port. Then, the remaining boundaries on the exterior were set to be “Scattering Boundary Condition”. Two “Perfect Matched Layers” (PMLs) were added above and below this structure to eliminate the stray electromagnetic waves.

## Additional Information

**How to cite this article**: Wang, H. *et al.* Graphene Based Surface Plasmon Polariton Modulator Controlled by Ferroelectric Domains in Lithium Niobate. *Sci. Rep.* **5**, 18258; doi: 10.1038/srep18258 (2015).

## Supplementary Material

Supplementary Information
