# Supplementary material for: Graphene Based Surface Plasmon Polariton Modulator Controlled by Ferroelectric Domains in Lithium Niobate
Source: Sci Rep. 2015 Dec 14;5:18258. doi: 10.1038/srep18258 (PMC4677406; doi:10.1038/srep18258)
Supplement: Supplementary Information [file srep18258-s1.pdf]

Supplementary Information

Graphene Based Surface Plamson Polariton Modulator Controlled by Ferroelectric Domains in Lithium Niobate

Hao Wang, Hua Zhao, Guangwei Hu, Siren Li, Hang Su, Jingwen Zhang

1. Parameter and constant values in surface conductivity and permittivity calculations

Table S1 Parameter and constant values in surface conductivity calculation

Physical Quantities	Value	Unit
T	300	K
k_B	1.3806488×10^{-23}	m^2 kg/(s^2 K)
e	1.60217657×10^{-19}	C
$\hbar$	1.05457173×10^{-34}	m^2 kg/s
v_F	9.5×10^{5}	m/s
ε_0	$8.854187817\times10^{-12}$	F/m
μ	10000	cm^2/(V s)
Δ	0.34	nm
ε_r	2.5	1

2. Kubo formula and semi-classical model of graphene conductivity and permittivity

Surface conductivity[1-3] of a graphene while ignoring the impact of magnetic field

$$\sigma_S(\omega,\mu_c,\Gamma,T)=\frac{je^2(\omega-j2\Gamma)}{\pi\hbar^2}\left[\frac{1}{(\omega-j2\Gamma)^2}\int_0^\infty \varepsilon\left(\frac{\partial f_d(\varepsilon)}{\partial\varepsilon}-\frac{\partial f_d(-\varepsilon)}{\partial\varepsilon}\right)d\varepsilon+\int_0^\infty\left(\frac{f_d(\varepsilon)-f_d(-\varepsilon)}{(\omega-j2\Gamma)^2-4(\varepsilon/\hbar)^2}\right)d\varepsilon\right]$$

where $f_d(\varepsilon)$ is Fermi-Dirac distribution

$$f_d(\varepsilon)=\frac{1}{e^{(\varepsilon-\mu_c)/k_BT}+1}$$

Here, ω is angular frequency, μ_c is chemical potential (Fermi energy level), Γ is scattering rate, T is temperature and ε is energy.

The first term comes from intraband transition (electron-photon scattering process)

$$\sigma_{S,\text{intra}}=-\frac{je^2k_BT}{\pi\hbar^2(\omega-j2\Gamma)}\left[\frac{\mu_c}{k_BT}+2\ln(e^{\frac{-\mu_c}{k_BT}}+1)\right]$$

Under the following condition,

$$k_BT\ll\mu_c,\hbar\omega$$

the second term i.e., interband transition can be expressed as

$$\sigma_{S,\mathrm{inter}} = -\frac{e^2}{4\hbar}\left[\theta\left(\hbar\omega - 2|\mu_c|\right) + \frac{j}{\pi}\ln\left|\frac{2|\mu_c| - \hbar(\omega - j2\Gamma)}{2|\mu_c| + \hbar(\omega - j2\Gamma)}\right|\right]$$

For mid-infrared wavelengths where the intraband transition dominates, the conductivity can be simplified to a Drude-like form as

$$\sigma_{\mathrm{intra-simplified}} = \frac{i\mu_c e^2}{\pi\hbar^2(\omega + i\tau^{-1})}$$

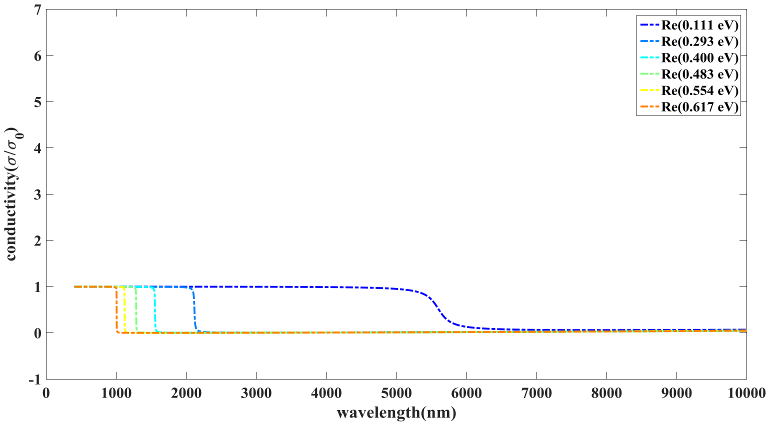

Figure S1 Real part of surface conductivity of monolayer graphene calculated by semi-classical model.

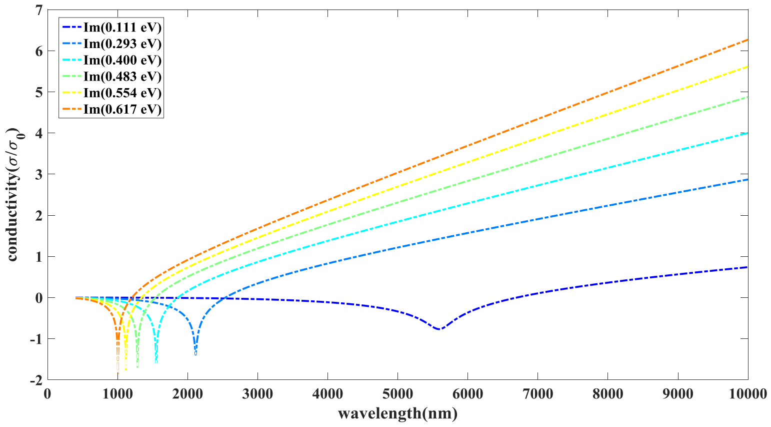

Figure S2 Imaginary part of surface conductivity of monolayer graphene calculated by semi-classical model.

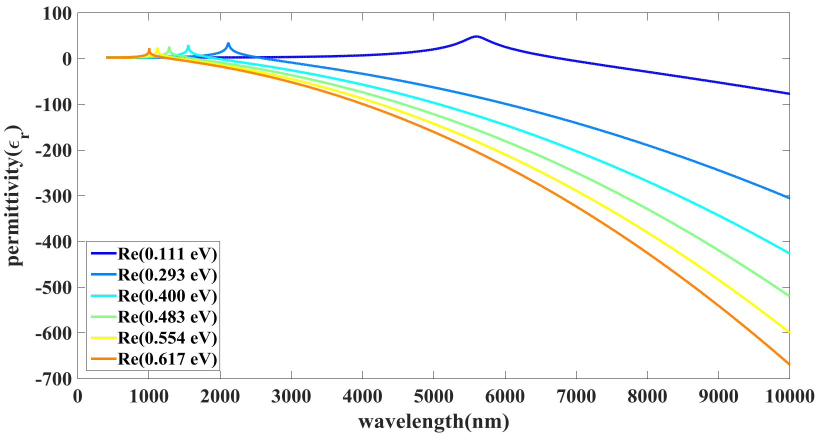

Figure S3 Real part of permittivity of monolayer graphene calculated by semi-classical model.

3. Spontaneous polarization along the *c* axis in $LiNbO_3$

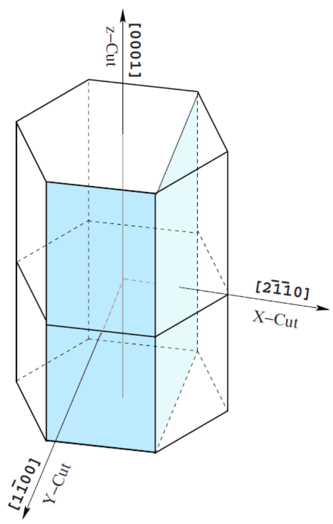

Fig. S4 Real-space representations of the x, y, z axes and of the X-cut,Y-cut and Z-cut (C-cut). [4]

Since the surface charge comes from the spontaneous polarization along the *c* axis in lithium niobate (Fig. S4), in our calculation we used the C-cut [0001] sample to realize chemical potential shift in graphene. Here we provide the spontaneous polarization values of lithium niobate obtained from both experiments and theoretical calculations, as shown in Table. S2, where P_S is the spontaneous polarization (upper limit), σ_S is the surface charge density, LDA is local-density approximation, GGA is generalized gradient approximation and DFT is density-functional theory. These results are consistent with the modern theory of polarization developed by Resta [10], King-Smith and Vanderbilt [11, 12]. Besides, the corresponding carrier concentrations are also presented. These results are regarded as the upper limit of spontaneous polarization values. The maximum chemical potential value 0.617 eV used in our calculation is reasonable for its corresponding surface charge density is $3.1\times10^{13}/cm^2$, which is one magnitude order smaller than the upper limit.

Table. S2 Spontaneous polarization of lithium niobate

Source	P_S (C/m^2)	σ_S (×10^{14}/cm^2)
Expt. [5]	0.71	4.44
Expt. [6]	0.70	4.38
Calc. [7]	0.77	4.81
LDA [8]	0.80	5.00
GGA [9]	0.80	5.00
DFT [10]	0.82	5.13

4. Parameter values used in refractive indexes calculation of $LiNbO_3$

Multi-oscillator models for the refractive index of oxide ferroelectrics such as $LiNbO_3$ have been widely used [13, 14]. A three-oscillator Sellmeier equation [15] coming from experimental data was used to calculate the CLN's anisotropic refractive indices,

$$n^2 - 1 = A\lambda^2 / (\lambda^2 - B) + C\lambda^2 / (\lambda^2 - D) + E\lambda^2 / (\lambda^2 - F)$$

where, n is refractive index, λ is wavelength (μm), the capital letters used in the equation are fitting coefficients (unit for B, D, F is μm^2). This equation incorporates two UV oscillators (the first and second terms) and also an IR oscillator (the last term) to account for the refractive-index behavior near the IR edge of the transmission range [16, 17]. According to the reviewer's advice, the coefficients have been rewritten into matrix **S**, for CLN refractive indices (n_e and n_o), the corresponding matrixes are $\mathbf{S}(n_e)$ and $\mathbf{S}(n_o)$ [15], respectively.

$$\mathbf{S} = \begin{bmatrix} A \\ B \\ C \\ D \\ E \\ F \end{bmatrix}, \quad \mathbf{S}(n_e) = \begin{bmatrix} 2.9804 \\ 0.02047 \\ 0.5981 \\ 0.0666 \\ 8.9543 \\ 416.08 \end{bmatrix}, \quad \mathbf{S}(n_o) = \begin{bmatrix} 2.6734 \\ 0.01764 \\ 1.2290 \\ 0.05914 \\ 12.614 \\ 474.6 \end{bmatrix}$$

5. Definition of factors for evaluating modulator

A) The signal extinction per unit length is defined as [18]

$$E_L = 10\lg\left(\frac{P_0}{P}\right) / L$$

where, P_0 is the power of incident light and P is the transmitted power in Air/Graphene/CLN modulator through length of a ferroelectric domain period. The whole domain is uniformly polarized in down direction.

B) The logarithmic extinction ratio per unit length (modulation depth) can be expressed as

$$M_L = 10\lg(\frac{P_{on}}{P_{off}})$$

where, P_{on} is the power at the output end of a ferroelectric domain period, should have the same value with P. P_{off} is similar except that the ferroelectric domain period has a down polarized domain and

a depolarized domain, which can be illustrated in Fig. S5.

C) The figure of merit (FoM) was defined as

$$FoM = \frac{M_L}{E_L} = \frac{|\alpha_{off} - \alpha_{on}|}{\alpha_{off}}$$

where, α_{off} and α_{on} are plasmonic amplitude absorption coefficients similar to the coefficients used in traditional electro-optic modulator, representing voltage-off and voltage-on states respectively. Here we can define them as domain-off and domain-on states. FoM describes the modulation ability of the domain-off in comparison to the domain-on state in length of a ferroelectric domain period.

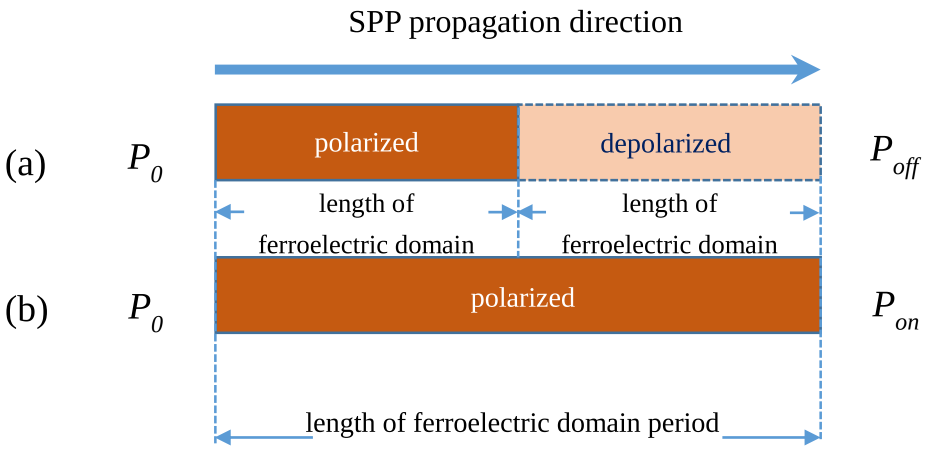

Figure S5 Lengths of ferroelectric domain and ferroelectric domain period.

Reference

[1]. Falkovsky, L. & Pershoguba, S. Optical far-infrared properties of a graphene monolayer and multilayer. *Phys. Rev. B* **76**, 153410 (2007).

[2]. Falkovsky, L. in *Journal of Physics: Conference Series.* 012004 (IOP Publishing, 2008).

[3]. Sernelius, B. E. Core-level spectra from graphene. *Phys. Rev. B* **91**, 045402 (2015).

[4]. Sanna, S. & Schmidt, W. G. Lithium niobate X-cut, Y-cut, and Z-cut surfaces from ab initio theory. *Phys. Rev. B* **81**, 214116 (2010).

[5]. Wemple, S., DiDomenico Jr, M. & Camlibel, I. Relationship between linear and quadratic eletro-optic coefficients in $LiNbO_3$, $LiTaO_3$, and other oxygen-octahedra ferroelectrics based on direct measurement of spontaneous polarization. *Appl. Phys. Lett.* **12**, 209-211 (1968).

[6]. Glass, A. & Lines, M. Low-temperature behavior of spontaneous polarization in $LiNbO_3$ and $LiTaO_3$. *Phys. Rev. B* **13**, 180 (1976).

[7]. Hafid, L. & Michel-Calendini, F. Electronic structure of $LiNbO_3$: densities of states, optical anisotropy and spontaneous polarisation calculated from the Xα molecular orbital method. *J. Phys. C: Sol. Stat. Phys.* **19**, 2907 (1986).

[8]. Veithen, M. & Ghosez, P. First-principles study of the dielectric and dynamical properties of lithium niobate. *Phys. Rev. B* **65**, 214302 (2002).

[9]. Sanna, S. *et al.* in *High Performance Computing in Science and Engineering '14* 163-178 (Springer, 2015).

[10]. Resta, R. Macroscopic polarization in crystalline dielectrics: the geometric phase approach. *Rev.*

Mod. Phys. **66**, 899 (1994).

[11]. King-Smith, R. & Vanderbilt, D. Theory of polarization of crystalline solids. *Phys. Rev. B* **47**, 1651 (1993).

[12]. Vanderbilt, D. & King-Smith, R. Electric polarization as a bulk quantity and its relation to surface charge. *Phys. Rev. B* **48**, 4442 (1993).

[13]. Zelmon, D. E., Small, D. L. & Jundt, D. Infrared corrected Sellmeier coefficients for congruently grown lithium niobate and 5 mol.% magnesium oxide–doped lithium niobate. *J. Opt. Soc. Am. B* **14**, 3319-3322 (1997).

[14]. Schlarb, U. & Betzler, K. Refractive indices of lithium niobate as a function of wavelength and composition. *J. Appl. Phys.* **73**, 3472-3476 (1993).

[15]. Schlarb, U. & Betzler, K. Refractive indices of lithium niobate as a function of temperature, wavelength, and composition: A generalized fit. *Phys. Rev. B* **48**, 15613 (1993).

[16]. Axe, J. & O'kane, D. Infrared dielectric dispersion of $LiNbO_3$. *Appl. Phys. Lett.* **9**, 58-60 (1966).

[17]. Barker Jr, A. & Loudon, R. Dielectric Properties and Optical Phonons in $LiNbO_3$. *Phys. Rev.* **158**, 433 (1967).

[18]. Babicheva, V. E. & Lavrinenko, A. V. in *SPIE Photonics Europe.* 842413 (International Society for Optics and Photonics, 2012).
